# C-Reactive Protein: Clinical and Epidemiological Perspectives

**DOI:** 10.1155/2014/605810

**Published:** 2014-02-06

**Authors:** Juan Salazar, María Sofía Martínez, Mervin Chávez, Alexandra Toledo, Roberto Añez, Yaquelín Torres, Vanessa Apruzzese, Carlos Silva, Joselyn Rojas, Valmore Bermúdez

**Affiliations:** ^1^Endocrine-Metabolic Research Center, “Dr. Félix Gómez”, Faculty of Medicine, University of Zulia, Maracaibo 4004, Zulia, Venezuela; ^2^Institute of Clinical Immunology, University of Los Andes, Mérida 5101, Mérida, Venezuela

## Abstract

An important etiopathogenic component of cardiovascular disease is atherosclerosis, with inflammation being an essential event in the pathophysiology of all clinical pictures it comprises. In recent years, several molecules implicated in this process have been studied in order to assess cardiovascular risk in both primary and secondary prevention. C-reactive protein is a plasmatic protein of the pentraxin family and an acute phase reactant, very useful as a general inflammation marker. Currently, it is one of the most profoundly researched molecules in the cardiovascular field, yet its clinical applicability regarding cardiovascular risk remains an object of discussion, considered by some as a simple marker and by others as a true risk factor. In this sense, numerous studies propose its utilization as a predictor of cardiovascular risk through the use of high-sensitivity quantification methods for the detection of values <1 mg/L, following strict international guidelines. Increasing interest in these clinical findings has led to the creation of modified score systems including C-reactive protein concentrations, in order to enhance risk scores commonly used in clinical practice and offer improved care to patients with cardiovascular disease, which remains the first cause of mortality at the worldwide, national, and regional scenarios.

## 1. Introduction

Owing to profound research and identification of the etiopathogenic basis of cardiovascular disease (CVD), as well as the diverse mechanisms implicated in the onset and progression of atherosclerosis [1], current studies in this area focus in the characterization of biomarkers for the early detection of the inflammatory activation underlying this process. In turn, this has improved methods for the estimation of cardiovascular risk (CVR), atherosclerotic plaque rupture, and even subsequent ischemic events [2], by establishing revised initial management measures based on the newfound greater knowledge on this group of ailments. Likewise, these molecules may complement the predictive ability of many classic CVD risk factors and serve as valuable prognostic information for several associated adverse effects [3, 4].

The biomolecule with the greater body of research both from a molecular and epidemiological perspective is C-reactive protein (CRP), a plasma protein of the pentraxin family and an acute phase reactant, which displays high sensitivity as a general inflammation marker [5]. Numerous studies have demonstrated the active participation of this molecule in the atherogenic process [6], and due to the discovery of high-sensitivity techniques for its determination, its stable plasmatic concentrations, and its relatively low costs, it may be of great use in the identification of patients at high risk, as a prognostic indicator and even as a therapeutic target in large populations [7].

Studies surrounding this molecule started over a decade ago, statistically associating the onset of coronary events, such as myocardial infarction (MI) and angor pectoris, with elevated serum levels of this protein [8]. From this point, experimental and epidemiological research [9, 10] has aimed at the elucidation of the true effect and predictive power of CRP in CVD, for both primary and secondary prevention, as an element to consider in equations for the calculation of absolute CVR in specific populations and as a parameter to be evaluated in all patients with an acute coronary event and patients undergoing therapeutic procedures in this field [11].

In this scenario, it is necessary to consider the diverse perspectives outlined worldwide and assess opinions for and against this controversial protein, in order to ultimately analyze the inclusion of its routine determination as part of the diagnostic work-up in patients with CVD in our population.

## 2. Cardiovascular Risk: Current Tendencies

According to the *Framingham Heart Study*, CVD comprises a broad spectrum of pathologies including coronary disease (MI, coronary insufficiency, and angor pectoris), cerebrovascular disease (ischemic ictus, hemorrhagic ictus, and transient ischemic events), peripheral artery disease (intermittent claudication), and heart failure (HF) [12–14]. For the clinical-epidemiological assessment of CVD, several studies [15, 16] have proposed the implementation of concepts such as absolute cardiovascular risk (aCVR), also known as global or total risk, which represents the probability of developing one of these disorders in a given period of time [17], and relative cardiovascular risk (rCVR), which accounts for the probability of developing a cardiovascular event in subjects with certain risk factors compared with individuals of the same age and gender without such factors [12].

The first great-scale epidemiologic study for the determination of the main causes of CVD was the *Framingham Heart Study* [18]. The algorithms resulting from this research have been validated and widely utilized in clinical practice for the estimation of aCVR in various populations worldwide [12], since they allow for risk stratification. These equations are based in cardiovascular risk factors (CRF) such as hypertension (HT), diabetes mellitus (DM), and dyslipidemia, among others. Nevertheless, results obtained from the application of these equations are not applicable in certain demographies, such as Spain, Italy, China, and Chile [19–21], where the risk of developing CVD is relatively low.

Indeed, although scoring systems for the estimation of aCVR are important tools, they display several limitations: modifications in long-standing population-specific patterns of disease onset—upon which CVR is estimated—may distort their clinical application. Moreover, most of these algorithms do not evaluate CVD in a global manner, but they are formulated to predict specific isolated components of it, especially coronary disease (CD) [17, 22–25], in opposition to the multifactorial concept of the etiology to which these equations for estimated aCVR are geared for [12, 18].

## 3. Cardiovascular Risk Factors: Classic versus Novel (Table 1)

A risk factor is a measurable element or characteristic that shares a causal relationship with an increment in the onset frequency of a given disease, constituting a significant independent predictive component for the risk of the developing this condition [18]. CRF have been described since the 1930s, reaching widespread notoriety in 1948 with the initiation of the *Framingham Heart Study*, which years later would define what are nowadays considered classic risk factors, in consonance and corroboration with other great epidemiologic studies [43, 44].

The most common classification of CRF labels them as either modifiable or nonmodifiable. Nonmodifiable CRF include age (men >45 years and women >55 years or menopausal), race (black), gender (male), and genetics (family history of CVD), while modifiable CRF cover obesity, HT, dyslipidemia, smoking, DM, and a sedentary lifestyle [22].

Throughout the years, interest has focused on the improvement of CVR assessment, given the rising incidence of CVD. This has led to the realization of numerous multidisciplinary studies linking the origin and evolution of CVD with new biochemical markers. These biomarkers have displayed an important predictive ability for the onset of cardiovascular events, as they have been shown to be closely related to the pathophysiological mechanisms of these diseases, with both local and systemic implications [45, 46]. Each of these molecules exhibits distinct biochemical features, including factors associated with low-grade inflammation (CRP, vascular adhesion molecules, interleukins, pentraxins, leukocytes, etc.) [31, 33, 47–49], components of the lipid profile (lipoprotein[a], apolipoproteins, triacylglycerides, etc.) [29, 50, 51], and elements linked to prothrombotic states (fibrinogen, homocysteine, D-dimer, etc.) [35, 37, 52].

From this diverse assortment of candidates, CRP appears to be the most profoundly studied regarding inflammation in the cardiovascular field [53]. Not only has CRP been associated with the chain of events occurring at the endothelium within the atherosclerotic process [54, 55], but it has also been linked with a direct, independent association with future cardiovascular events in several great-scale epidemiologic reports [27, 56, 57], as well as with other disorders of metabolic origin, both in adult and pediatric populations [58, 59].

## 4. Clinical Applicability of C-Reactive Protein in Risk Assessment

### 4.1. C-Reactive Protein as a Cardiovascular Risk Factor

Kroop and Shackman [60] were the first to report alterations of CRP levels in patients with MI. Three years later, Gurevin and Kozonis [61] proposed this protein as a reflection of the natural history of this disorder, but it was only in the mid-1990s that research by Ridker et al. [62] suggested this molecule to acquire greater relevance within the group of novel risk factors, after closely linking it not only with MI but also with cerebrovascular events.

Currently, it is well known that CRP levels may rise due to a several processes of inflammatory etiology (Table 2). This lack of specificity may concern many physicians when assessing CRP in the clinical scenario. However, methods for the quantitative determination of this acute phase reactant have reached detection thresholds lower than 1 mg/L [63], with a mean serum concentration of 0.8 mg/L in young adults with no underlying inflammatory processes [5]. These newer quantification techniques have been dubbed “high-sensitivity” and are essential for the consideration of this protein as a CRF. Initially, high-sensitivity quantification methods were based on ELISA, utilized in several population studies despite its cumbersome routine use in clinical laboratories [64]. As a consequence, more accessible methods, such as immunonephelometric techniques [65] and, more recently, automatized immunoluminometry and immunoturbidimetry, have been implemented, improving the sensitivity of the quantification even in cases of very low concentrations [66]. Additionally, these are inexpensive techniques, an important aspect regarding its routine use in clinical practice [63].

**Figure 1:**
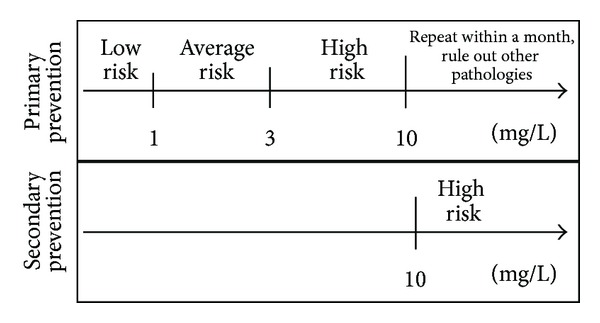
Use of high-sensitivity CRP levels for the stratification of cardiovascular risk (primary prevention) and as a prognostic factor in acute coronary syndrome (secondary prevention) [69, 70].

In light of these advantages and findings from several epidemiological studies, the Center for Disease Control and Prevention (CDC) and American Heart Association (AHA) established in 2003 the first guidelines for the interpretation of markers of CVR, with special emphasis on the use of CRP in primary prevention. In addition, the distribution of CRP serum concentrations in tertiles was presented: first tertile: <1 mg/L, second tertile: 1–3 mg/L, and third tertile: >3 mg/L. This model is widely accepted in clinical practice given that, after adjustment for other CRF, subjects with CRP levels between 1 and 3 mg/L had a 50% greater CVR than those with concentrations <1 mg/L. Likewise, individuals with levels >3 mg/L had a CVR approximately twice as high as those with values <1 mg/L. With this outline, the CDC designated subjects within the first tertile as low risk, those in the second tertile as average risk, and those in the third tertile as high risk of developing CVD [67, 68]. The utilization of CRP in the assessment of patients with CVD must be accompanied by a detailed clinical record and an adequate interpretation in order to avoid false positives. Measurement of CRP should be omitted if infection is suspected, or if there is history of a traumatic event within the previous 2 weeks. If concentrations >10 mg/L are obtained, the measurement should be repeated, and 2 subsequent determinations should be realized with an interval of 1 month, selecting the lowest value returned for the assessment of CVR [63, 68, 69].  Figure 1 displays the recommended scheme for the interpretation of CRP levels for CVR stratification.

CRP cut-off values have been proposed for the evaluation of patients in 3 distinct clinical conditions: apparently healthy subjects, stable patients with CVR or diagnosed CVD, and patients with acute coronary syndrome (ACS) [2]. Nonetheless, the first scenario is particularly important, considering that the estimation of aCVR is pivotal for the initiation of preventive and therapeutic management. For example, individuals with a high risk as estimated by the equations from the *Framingham Heart Study* (entailing >20% risk of developing CD in 10 years) are indicated to receive intensive medical intervention and/or pharmacologic therapeutic management. The latest guidelines published by the National Academy of Clinical Biochemistry (NACB) recommend the quantification of CRP in patients with moderate risk (10–20% risk of developing CD in 10 years by the Framingham classification) and in patients that raise doubts regarding their management. These criteria allow for the evaluation of the application of intensive therapeutic measures including both lifestyle modifications and pharmacotherapy, with the quantification of CRP in primary attention being classified as a Class Ia indication [68, 70].

The importance of CRP assessment prompted Ridker et al. [74] to develop, validate, and demonstrate a project named the “*Reynolds Risk Score*,” a high-precision predictive model for CVR in 10 years, initially created for women and later for men [75], which included the use of high-sensitivity CRP and family history of CD within its clinical algorithm, along with traditional risk factors: age, blood pressure, smoking, total cholesterol, and HDL-C concentration, allowing for the reclassification of women and men in distinct CVR categories. A meta-analysis of over 50 studies realized by Kaptoge et al. [76] revealed CRP concentrations to have a strong association with mortality of vascular origin, as well as with risk of CD and ischemic ictus. Nevertheless, this association with ischemic vascular disease may depend fundamentally on conventional risk factors.

### 4.2. C-Reactive Protein in Acute Coronary Events

Similar to recommendations with respect to primary prevention, the AHA and CDC suggest the determination of high-sensitivity CRP serum concentrations in patients with SCA, with a cut-off of >10 mg/L as a predictive factor of subsequent acute events [68, 69]. Indeed, these standard have been recently ratified by several clinical guidelines and population studies [70, 78].  Figure 1 shows the CRP cut-off values used in both primary and secondary prevention.

Indeed, research has covered the analysis of SCA patients both with and without ST segment elevation [79, 80], with the fundamental objective of identifying subjects at high risk for recurrence of these events and death [81]. On the other hand, its utilization has also been suggested as a prognostic marker in mid- and long-term after an acute event, independently of other markers such as troponins and B-type natriuretic peptide. In this sense, the joint analysis of Troponin-CRP is a very useful assessment method given the additive behavior of both biomarkers [82]. This practice would enhance the identification of patients with a high-risk prognosis and the necessity of intervention or “aggressive” monitoring [67]. Nonetheless, efforts are still directed to irrefutably demonstrate that this molecule improves risk estimation in all patients [83].

Therefore, novel research, such as that proposed by Schiele et al. [86], aims to determine the predictive value of the “Global Registry of Acute Coronary Events” (GRACE), a clinical scale designed for the estimation of mortality risk or HF risk in patients with SCA, with the inclusion of CRP serum concentrations in the model. Results have demonstrated that the addition of this molecule improves the classification of these patients in distinct risk categories [86]. Tables 3 and 4 summarize studies analyzing CRP for both primary and secondary prevention.

## 5. Is CRP a Risk Marker or a Risk Factor?

**Figure 2:**
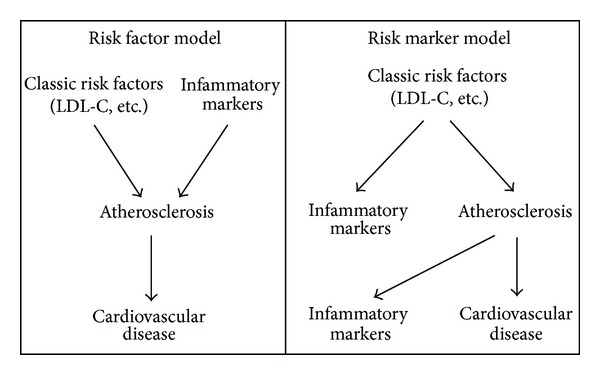
Models used to ascertain the role of an inflammatory marker.

According to official definition of the United States National Institute of Health (NIH), a biomarker is “a characteristic that is objectively measured and evaluated as an indicator of normal biological processes, pathogenic processes, or pharmacologic responses to a therapeutic intervention” [87]. The association with any given disease may be exclusively statistical and it does not require an established causal relationship. Meanwhile, a risk factor is associated with a given pathology due to its participation in the etiopathogenic mechanisms triggered by itself [88] (Figure 2).

Regarding CRP, a controversial worldwide debate persists over its participation in CVD. Several retrospective and prospective studies, as well as meta-analyses, have described an association with MI [71, 72, 84, 89, 90], coronary insufficiency [4, 73, 91], cerebrovascular disease [85], and peripheral artery disease [92], behaving as an important mortality predictor [69]. As such, a large portion of scientific opinion supports CRP as a risk marker, in response to the inflammatory process within the atherosclerotic plaque and other previously established CRF [88]. Conversely, its role as a CRF is heavily debated [77, 93, 94]. Despite the large body of evidence associating CRP with atherosclerotic lesions, the lack of a direct correlation between its concentration and the extension of atherosclerosis as determined by imaging techniques constitutes one of the main arguments for those who oppose this much-disputed mechanism [88], along with its well-known associations with other risk factors included in the Framingham equations [95]. However, we consider there is sufficient evidence that shows an intimate association between this molecule and cardiovascular events, both at a clinical and molecular level, granting its consideration not only as a biomarker, but also as a true risk factor [96].

Likewise, various reports have demonstrated that initiation of pharmacologic treatment prompted by elevated CRP levels may lower the onset of coronary events both in primary and secondary prevention, as suggested by the AirForce/Texas Coronary Atherosclerosis Prevention Study (AFCAPS/TexCAPS), wherein the administration of lovastatin reduced the frequency of coronary events in patients under primary prevention with low/normal LDL-C levels and high CRP concentrations [97]. Similarly, secondary prevention studies such as the Reversal of Atherosclerosis with Aggressive Lipid Lowering (REVERSAL) study show the use of higher statin doses (Atorvastatin 80 mg versus Pravastatin 40 mg) to significantly lower both LDL-C and CRP levels after 18 months of therapy. Amelioration of atherosclerotic progression was also found as assessed by intravascular ultrasound, with a significant independent correlation between decreased CRP and atherosclerotic progression [98]. These results, in addition to others such as the JUPITER study [99], demonstrate the usefulness of CRP for the identification of subjects in risk, and they also hint towards its potential role as a therapeutic target in the atherosclerotic process [100]. Therefore, future research should continue to more thoroughly study the effects of the reduction of serum CRP levels.

## 6. Conclusions

Given the ever-increasing problematic that CVD represents nowadays worldwide, it is necessary to exhaustively evaluate all subjects who may develop them. This possibility may be measured or quantified through scoring systems created based on the *Framingham Heart Study*, which allows for the calculation of absolute risk of developing a cardiovascular event based on risk factors inherent to each studied individual. Traditional risk factors are proved predictive elements for the development of disease that share a causal relationship with it. In the case of CVD, these include age, gender, obesity, HT, dyslipidemia, smoking, DM, and a sedentary lifestyle, among others.

The designation of new risk factors stemming from advances in the comprehension of the inflammatory physiopathology of CVD has led research to try and elucidate which of these novel and emergent elements display all required criteria to be considered true risk factors, and which have solely exhibited a casual statistical association. C-reactive protein is one of the numerous molecules that fit this description, but its properties and features have led it to become one of the main targets for researchers worldwide. Its utilization in the clinical setting is still discussed by a myriad of organizations, as well as its role in CVD. In this aspect, CRP is more than a simple biomarker and current findings tightly link this protein with the atheromatous plaque. Its implementation is based on the guidelines suggested by the NACB, which delimit its application to a certain population at risk, and sets a cut-off point for its serum levels.

However, many aspects still remain to be elucidated, requiring the assessment of CRP behavior across ethnic groups (Asians, Africans, and Hispanics) since most studies have been limited to European and North American cohorts. Likewise, further research would clarify the true role of CRP in the development of CVD, establishing its relative importance regarding other CRF, which is a particularly relevant aspect given the potential benefit that may be rendered by broadening the spectrum of variables included in CVR-estimating calculation tools [101].

Furthermore, research should be expanded to further age groups, analyze the impact of CRP in coronary event prognosis, and decipher the phenomena linking it to the atherogenic process, in order to exploit its potential efficacy as a therapeutic target [102]. The answers to these matters would allow the confirmation of the feasibility of CRP quantification and the formulation of management guidelines for our patients, based in the measurement and the clinical picture of each individual.

It should be noted that despite the high prevalence of CVD in our population, CRP quantification remains a nonroutine procedure, neither in primary attention nor in specialist management. Therefore, raising awareness among all health personnel represents a fundamental basis for this kind of studies, which should be readily spread in order to offer a more efficient management to our patients. This is especially relevant for population reports such as our local study, which shows a particular behavior for CRP, with levels differing from those of most other worldwide studies—with lower average serum concentrations—as well as a close association with the metabolic syndrome, especially with high triacylglyceride levels and elevated waist circumference values (with cut-off points higher than those proposed by all current consensuses) [103]. These findings demonstrate the need to evaluate the behavior of these metabolic variables in each region in order to assess the comparative influence these cardiometabolic alterations exert over CVR and to set management guidelines adapted to each specific cohort.

## Figures and Tables

**Table 1:** Risk factors for cardiovascular disease.

Classic factors (reference)	Novel factors (reference)
(i) Age [26]	(i) C-reactive protein [27]
(ii) Family history [28]	(ii) Lipoprotein[a] [29]
(iii) Race [30]	(iii) Pentraxin 3 [31]
(iv) Arterial hypertension [32]	(iv) Leukocytes CD31+ [33]
(v) Diabetes mellitus [34]	(v) Homocysteine [35]
(vi) Smoking [36]	(vi) Fibrinogen [37]
(vii) Dyslipidemia [38]	(vii) Adiponectin [39]
(viii) Obesity [40]	(viii) Alcohol [41]
(ix) Sedentary lifestyle [42]	

**Table 2:** C-Reactive Protein in certain pathologies.

Acute phase response with high CRP release	
Infection	Bacteria, mycobacteria, viruses, and fungi
Postinfectious allergic complications	Rheumatoid arthritis and erythema nodosum
Inflammatory diseases	Crohn's disease, psoriatic arthritis, systemic vasculitis, and Reiter's disease
Necrosis	Myocardial infarction and acute pancreatitis
Trauma	Surgeries, fractures, and burns

Acute phase response with low CRP release	

Systemic lupus erythematosus, scleroderma, ulcerative colitis, and dermatomyositis	

**Table 3:** Studies on cardiovascular risk assessment utilizing C-reactive protein.

Author (reference)	Sample	Results
Ridker et al. [62]	543 apparently healthy men	Serum CRP levels predict MI and CVD: The quartile with the highest CRP levels had a greater risk of MI (RR: 2.9; *P* < 0.001) and CVD (RR: 1.9; *P* < 0.02) than the lowest quartile.

Ridker et al. [71]	366 apparently healthy women (122 developed a cardiovascular event)	Serum CRP levels of patients who had a cardiovascular event were higher than control patients (*P* = 0.0001). Patients with the highest levels had a greater risk of developing MI or CVD (RR: 7.3; *P* = 0.0001).

Ridker et al. [72]	27939 apparently healthy women	CRP is a more powerful predictor of cardiovascular events than LDL-C. The RR for a first cardiovascular event was, according to CRP quintiles: 1.4–1.6–2.0–2.3 (*P* < 0.001).

Cesari et al. [73]	2225 participants aged 70–79 years without previously diagnosed cardiovascular disease	Inflammatory markers are predictors of cardiovascular events in elderly patients. CRP was associated with CHF (RR: 1.48; IC: 1.23–1.78).

Ridker et al. [74]	24558 initially healthy women (≥45 years of age)	2 new algorithms were developed for the calculation of global cardiovascular risk, reclassifying a great part of women with average risk according to conventional scoring systems.
Ridker et al. [75]	10724 initially healthy women (≥50 years of age)	A new prediction model was developed for the calculation of global cardiovascular risk, including CRP and family history of cardiovascular events. Over 20.2% of the population was reclassified from the original distribution of conventional scoring systems.

Kaptoge et al. [76]	160309 subjects without history of vascular disease (54 prospective studies)	The association of CRP with vascular disease depends on other inflammatory markers and classic risk factors. After multiple adjustments, the RR for coronary disease was (1.23; IC: 1.07–1.42), for CVD (1.32; IC: 1.18–1.49), and for vascular cause mortality (1.34; IC: 1.18–1.52).

Maiorana et al. [77]	37 patients with 3 or more cardiovascular risk factors	10 patients had LDL-C >100 mg/dL, fibrinogen >350 mg/dL, and CRP >2.6 mg/L; 6 of these patients presented a positive ischemia by exercise testing and coronary disease.

CRP: C-reactive protein; MI: myocardial infarction; CVD: cerebrovascular disease; RR: relative risk; LDL-C: low-density lipoproteins; CHF: congestive heart failure; CI: confidence interval.

**Table 4:** Studies on acute coronary syndrome prognosis utilizing C-reactive protein.

Author (reference)	Sample	Results
Pietila et al. [84]	188 patients with MI	Serum CRP levels in patients with MI predict mortality up to 6 months after the event. Highest levels were found between the 2nd and 4th days after infarction, the highest mean concentration being 65 mg/L; IC: 58–71 in patients who survived 24 months.

Gussekloo et al. [85]	245 patients (80 deceased due to CVD after 5-year follow-up)	CRP is a powerful yet unspecific risk factor for CVD in the elderly. Serum CRP levels of those who died due to CVD were twice as high than those of control subjects (5.7 mg/L versus 2.7 mg/L; *P* < 0.005).

Mueller et al. [79]	1042 ACS patients without ST segment elevation	CRP is an independent predictor of mortality short- and long-term in ACS patients without ST segment elevation who received early invasive treatment. In-hospital mortality was 1.2% in patients with (<3 mg/L), 0.8% (1–3 mg/L), and 3.7% (>10 mg/L), with RR = 4.2 for mortality.

Morrow et al. [78]	3813 ACS patients	After multiple adjustments, patients with serum CRP levels 1–3 mg/L had a greater mortality risk (HR: 2.3; IC: 1.2–4.6) in comparison with those with levels <1 mg/L. The mortality risk for patients >3 mg/L was even higher (HR: 3.7; IC: 1.9–7.2).

Schiele et al. [86]	1901 ACS patients	CRP is modest yet independent predictor of mortality within the first month after ACS. Subjects with levels >22 mg/L (4th quartile) had 4 times greater mortality risk within 30 days.

Caixeta et al. [80]	2974 ACS patients	Patients with the highest serum CRP levels (4th quartile) presented a greater mortality risk within 30 days in comparison to the 1st quartile (2.3 versus 1.3%; *P* = 0.0004), as well as within a year after the event (5.5 versus 2.8%; *P* = 0.0003).

CRP: C-reactive protein; MI: myocardial infarction; CI: confidence interval; CVD: cerebrovascular disease; ACS: acute coronary syndrome; RR: relative risk; HR: hazard ratios.
